# The diagnostic and prediction performance of MR diffusion kurtosis imaging in the glioma molecular classification: a systematic review and meta-analysis

**DOI:** 10.3389/fneur.2025.1543619

**Published:** 2025-04-25

**Authors:** Hongfang Zhao, Zonggang Hou, Qifeng He, Xinlong Liu, Jian Xie

**Affiliations:** ^1^Department of Neurosurgery, Beijing Tiantan Hospital, Capital Medical University, Beijing, China; ^2^Beijing Tiantan Hospital, Capital Medical University, Beijing, China

**Keywords:** glioma genotype, molecular diagnosis, DKI, diagnostic accuracy, meta-analysis

## Abstract

**Background:**

Although diffusion magnetic resonance imaging (dMRI), particularly diffusion kurtosis imaging (DKI), has demonstrated efficacy in distinguishing between low- and high-grade gliomas, its predictive utility across various molecular genotypes remains unclear. Evaluating the accuracy of DKI and identifying sources of heterogeneity in its predictive performance could advance noninvasive molecular diagnostic methods and support the development of personalized treatment strategies.

**Materials and methods:**

A literature search of the PubMed, Web of Science, Cochrane Library, Embase, and Medline databases was performed. The studies retrieved were screened by two researchers (HFZ and ZGH), and those fulfilling the inclusion criteria were subsequently included in the meta-analysis. Study quality was assessed using the Quality Assessment of Diagnostic Accuracy Studies 2 (QUADAS-2) tool. The analyses summarized the mean differences in mean kurtosis (MK) and mean diffusivity (MD) in patients harboring various genotypes using suitable models, and explored heterogeneity. Finally, a bivariate restricted maximum likelihood estimation method and meta-regression analysis were performed to assess diagnostic potential and stability.

**Results:**

Fourteen studies comprising 886 patients were included in this meta-analysis. Regarding MK and MD, the mean difference between isocitrate dehydrogenase (*IDH*) mutation and *IDH* wild type was −0.21 (95% confidence interval [CI] −0.27 to −0.15; *I*^2^ = 93%) and 0.22 (95% CI 0.11 to 0.33; *I*^2^ = 92%), respectively. This heterogeneity could be explained by imaging parameters such as repetition time, echo time, maximal *b*-value, and number of diffusion directions. However, the mean difference did not reflect the genetic status of 1p/19q, *α*-thalassemia/mental retardation syndrome-X-linked (*ATRX*) gene, or O_6_-methylguanine-DNA-methyltransferase (*MGMT*). Analysis of diagnostic accuracy revealed that the pooled areas under the curve for MK and MD, based on *IDH* status, were 0.96 (95% CI 0.93 to 0.97) and 0.76 (95% CI 0.71 to 0.81), respectively. Heterogeneity was not observed for these DKI parameters.

**Conclusion:**

MK and MD exhibited potential diagnostic utility in the prediction of glioma molecular status and should be explored in medical practice. These parameters should be compared with other MRI models to develop a stable and suitable genetic molecular prediction method for patients with gliomas.

**Systematic Review Registration:**

https://www.crd.york.ac.uk/PROSPERO/view/CRD42024568923, CRD42024568923.

## Introduction

**Figure 1:**
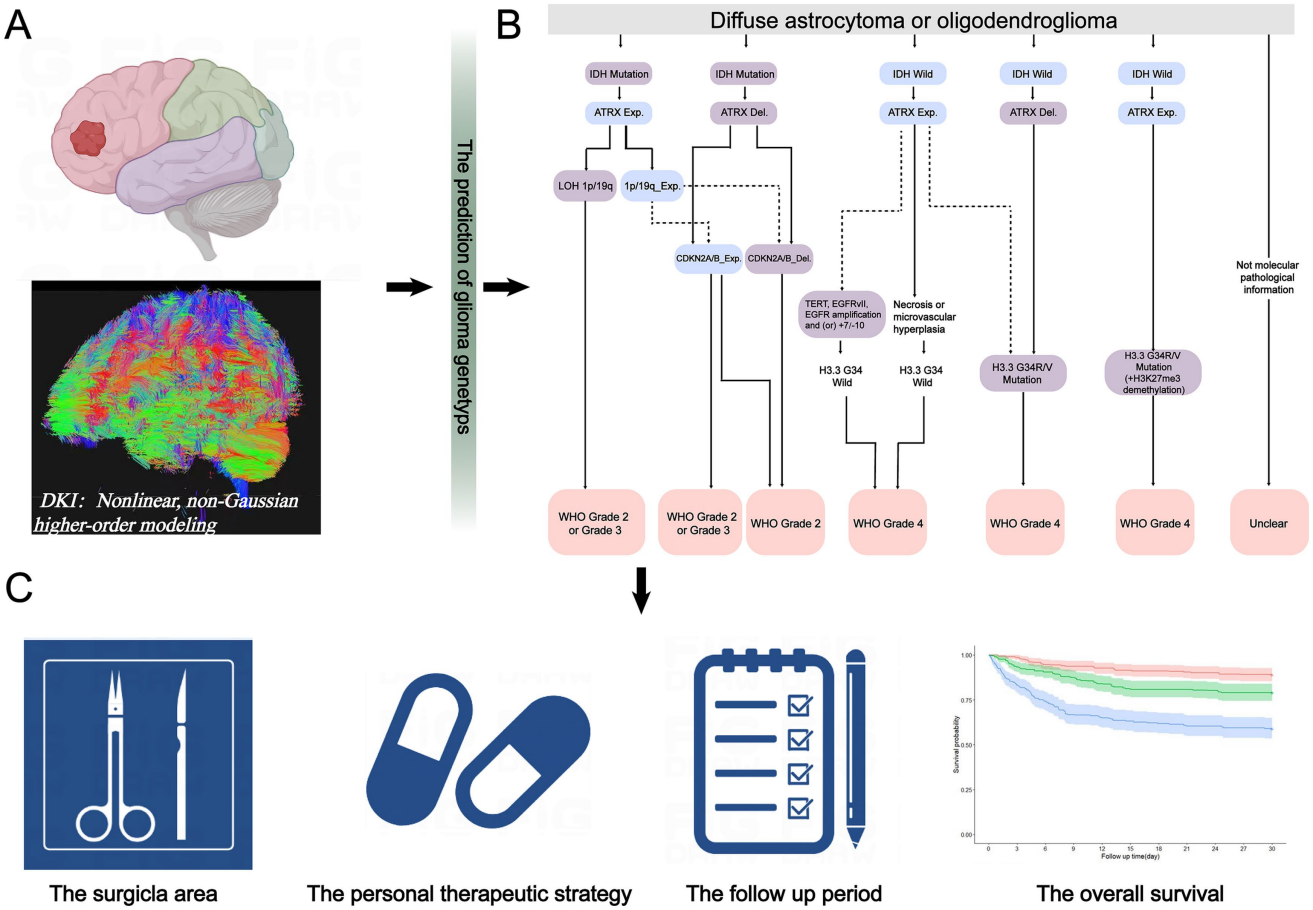
Significance of diffusion kurtosis imaging (DKI) and its key parameters in patient treatment. **(A)** When a patient was diagnosed with glioma, DKI was recommended. **(B)** Based on the molecular expression status predicted from the patient’s DKI main parameters’ values, clinicians could more accurately determine the histological grade. **(C)** Then, this information guided decisions on the extent of surgical area and the individualized adjuvant treatment strategies. Finally, patient survival rates could be monitored during follow-up, contributing to the advancement of medical care.

Gliomas are the most common primary intracranial malignancy (1). Clinicians have reported significant differences in treatment response, risk for recurrence, and overall survival among patients diagnosed with gliomas (2). Previous studies have suggested that these differences could be attributed to molecular expression status, especially isocitrate dehydrogenase (*IDH*), chromosomal alterations in *1p/19q*, alpha-thalassemia X-linked intellectual disability syndrome (*ATRX*), and O6-methylguanine-DNA methyltransferase (*MGMT*) (3). For example, Galbraith et al. (4) described the high heterogeneity and invasiveness of wild-type *IDH* glioma cell clusters. Similarly, Iwadate et al. (5) demonstrated that *1p/19q* co-deletion could reflect favorable outcomes and a lower risk for disease recurrence. Moreover, clinical trials have shown that some genetic markers—particularly *ATRX* and *MGMT*—were associated with therapeutic responses (5, 6). These findings highlight the importance of identifying the genetic status of gliomas to guide treatment strategies and improve patient outcomes (Figure 1). Traditionally, molecular diagnoses have primarily been determined from tissue samples obtained after surgical resection. However, not all patients accept the risk for neurological damage and mortality associated with surgery (7). Therefore, developing noninvasive diagnostic methods is critical for those who are unable or unwilling to undergo surgical intervention.

With advances and developments in medical technologies, diffusion magnetic resonance imaging (dMRI) and its related sequences, particularly diffusion-weighted imaging (DWI) and diffusion tensor imaging (DTI), have demonstrated potential for predicting glioma molecular subtypes based on the capture of water molecular condition (8–10). This non-invasive and specific technique has attracted the interest of clinicians and radiologists. Several trials have supported the predictive potential of dMRI for the molecular classification of gliomas. For example, Zhang et al. (9) analyzed preoperative MRI data from 247 patients diagnosed with gliomas and found that the apparent diffusion coefficient value on DWI could predict glioma histological grade and *IDH* expression status. Similarly, Xiong et al. (10) reported that fractional anisotropy values on DTI were significantly different between *IDH* mutant and *IDH* wild-type gliomas in 90 patients. However, due to volume and mass effects, the parameter values derived from DWI and DTI may be influenced by tumor heterogeneity and the surrounding normal peritumoral tissue. While it is possible to define and analyze the tumor region at the voxel level, the influences of regional overlap and tumor heterogeneity cannot be entirely eliminated. Similarly, due to the mass effect of gliomas, normal brain tissue, nerve fiber bundles, and small blood vessels may be displaced or distorted (11, 12). We have identified tumor regions as accurately as possible using manual drawing and software-based analyses. However, irrelevant brain structures cannot be completely recognized or eliminated based on imaging data (13). Therefore, basic dMRI sequences, such as DWI and DTI, may not accurately capture the complex information crucial for the prediction of genetic status (14). Additionally, magnetic resonance spectroscopy and amide proton transfer imaging techniques can assist clinicians in predicting the gene expression status of gliomas by analyzing metabolite concentration and chemical environment (15, 16). Volume and mass effects still influence MRI-related analyses. Therefore, it is necessary to develop higher-resolution MRI technologies.

Diffusion kurtosis imaging (DKI) can reveal the microstructural complexity of tumor tissues and provide additional metrics related to the non-Gaussianity of water diffusion (17). Previous studies have shown that DKI can effectively distinguish between low-grade gliomas (LGG) and high-grade gliomas (HGG) (18). Additionally, recent meta-analyses have further supported the predictive value of DKI in differentiating glioma grades (Table 1). For instance, meta-analyses conducted by Huang et al. (19) and Abdalla et al. (20) included 270 and 460 patients, respectively, both confirming the high diagnostic accuracy of DKI in glioma grading. Furthermore, Falk et al. (21) reported that mean kurtosis (MK), the main parameter of DKI, can differentiate HGG from LGG with a sensitivity of 0.85 and a specificity of 0.92. Meanwhile, Luan and his team found similar results (22). Among these studies, Xu and his colleagues conducted the most comprehensive meta-analysis to date, incorporating the largest number of studies and patients (23). Their findings provided strong evidence supporting the value of DKI in the prediction of glioma grades. Although DKI can predict glioma grades (HGG or LGG) stably, the utility of DKI for predicting glioma genotypes, like *IDH*, *ATRX*, *MGMT* genetic statuses, remains controversial. Su and Xu (24, 25) found that mean diffusivity (MD), a DKI parameter, could predict *IDH* mutations, with higher MD values observed in *IDH*-mutant gliomas. In contrast, Zhao et al. (26) reported that DKI parameters failed to provide consistent and reliable results for differentiating the *IDH* status. To address these conflicting results, we conducted a meta-analysis to assess the predictive utility of DKI. Our aim was to determine whether DKI could be considered a promising noninvasive diagnostic method, contributing to improved diagnostic accuracy and molecular classification in glioma management (27).

**Table 1:** Meta-analyses investigating Diffusion Kurtosis Imaging (DKI) to predict low (LGG) vs. high-grade glioma (HGG).

First author	Registered number/conducted data	Diagnostic methods	Studies number	Patients number (LGG/HGG)	The pooled efficacy	Publish bias	Key findings
Falk Delgado A.	CRD 42017064204	N/A	5	245 (108/137)	Sensitivity: 85% (95% CI, 74–92%) Specificity: 92% (95% CI, 81–96%) ROC curve: 0.94	Low	The DKI parameter MK had high accuracy in the discrimination between glioma grades. DKI could be added to the routine imaging protocol for work-up of suspected gliomas.
Xu C.	Up to December 15, 2020	Histopathology or Clinical diagnosis	13	706 (277/429)	Sensitivity: 88% (95% CI, 83–91%) Specificity: 91% (95% CI, 86–95%) ROC curve: 0.93 (95% CI, 0.90–0.95)	Low	DKI demonstrated a high diagnostic performance for differentiation of LGG from HGG.
Luan J.	Up to 2019.	MRI technology	16	675 (340/335)	Sensitivity: 88% (95% CI, 82–92%) Specificity: 95% (95% CI, 78–91%) ROC curve: 0.93 (95% CI, 0.91–0.95)	Low	Quantitative parameters of DKI, especially MK, had high diagnostic accuracy for preoperative grading of gliomas.
Abdalla G.	CRD 42018099192	Histologic and immunohistochemistry examination	9	460 (230/230)	Sensitivity: 87% (95% CI, 78–92%) Specificity: 85% (95% CI, 76–91%) ROC curve: 0.92	Low	DKI showed good diagnostic accuracy in the differentiation of HGG and LGG gliomas further supporting its potential role in clinical practice.
Huang R.	Up to April 31, 2018	MRI technology	5	270 (116/154)	Sensitivity: 91% (95% CI, 78–96%) Specificity: 91% (95% CI, 80–97%) ROC curve: 0.96	Low	This current meta-analysis provided evidences that DKI had the high diagnostic accuracy to differentiate HGG from LGG

## Methods

A team consisting of two neuro-oncologists, two neurosurgeons, and a senior statistician meticulously designed, registered, and conducted the meta-analysis in accordance with the Preferred Reporting Items for Systematic Reviews and Meta-Analyses (PRISMA) guidelines (Supplementary Tables S1, S2). The analysis was prospectively registered in PROSPERO on July 24, 2024 (CRD 42024568923) (28).

### Electronic search and eligibility criteria

An online literature search was conducted by a neuro-oncologist and a neurosurgical clinician (ZGH, with over 30 years of clinical experience, and HFZ, with 4 years of professional training as a neuro-oncologist) across five databases: PubMed, Web of Science, Cochrane Library, Embase, and Medline. The search covered the period from January 1, 1970, to July 25, 2024. The full search queries and step-by-step results are summarized in Supplementary Tables S3–S6. The inclusion criteria were as follows: (1) Histological grades and genotypes of gliomas must be identified through histopathological examination; (2) The included studies should provide detailed information on the patient population and the sensitivity and specificity rates of the diagnostic methods; (3) The DKI parameters must be derived from tumor tissue and include at least one of the following: MK or MD, along with the 95% confidence interval (95% CI); (4) The genetic status of patients reported in the article should encompass at least one of the following: *IDH*, *1p/19q*, *ATRX*, or *MGMT*; and (5) The included articles must ensure the application of contemporary imaging technologies. Both prospective and retrospective observational cohort studies were eligible for inclusion in this meta-analysis. Comments, conference abstracts, reviews, case reports, duplicate articles, or articles lacking DKI parameters were progressively excluded from the search.

### Data extraction and study quality assessment

To evaluate the diagnostic efficiency of DKI in glioma genotypes, a standardized table was established for data collection prior to the study. Two authors (HFZ and ZGH) independently performed a literature search and review, and documented the findings. Data extracted from the included studies were as follows: first author; publication year; trial type; population; number of patients with each genotype; mean age; World Health Organization criteria; histological grade; imaging vendors and strength; *b*-value and maximal *b*-value; repetition time; echo time; diffusion direction; MK; MD; and prediction efficacy (sensitivity and specificity). To mitigate selection bias, the reviewers compared results and analyzed any discrepancies at the conclusion of the extraction process. If contentious differences could not be reconciled, a senior researcher (JX) performed the second round of data extraction. The quality of the included studies was assessed using the Quality Assessment of Diagnostic Accuracy Studies-2 (QUADAS-2; Supplementary Tables S7, S8) (29).

### Statistical analysis

Mean differences in MK and MD were summarized to analyze variations across various glioma genotypes through inverse variance meta-analysis using an appropriate model. If preliminary heterogeneity was assessed as low or moderate (*I*^2^ ≤ 50%), a fixed effects model was applied; conversely, if heterogeneity was high (*I*^2^ > 50%), a random effects model was adopted (30). To explore sources of heterogeneity, further subgroup analyses based on patient characteristics, imaging vendors, and pre-processing parameters were performed. The results of the τ^2^ statistic and *χ*^2^ test were examined to evaluate variance between studies. The *I*^2^ statistic was also utilized to assess heterogeneity in the subgroup analyses. To estimate the diagnostic accuracy of DKI for glioma genotypes, a bivariate random effects meta-analysis with a restricted maximum likelihood estimation method was used. A likelihood ratio scattergram was used to assess model fit effects. Publication bias was examined using a funnel plot and the Deek’s test. A Fagan nomogram was used to indicate post-test probabilities based on varying pre-test probabilities for clinical decision making. Forest plots of diagnostic scores, odds ratio, positive diagnostic likelihood ratio (PLR), and negative diagnostic likelihood ratio (NLR) were used to quantify the diagnostic effect. Statistical analyses were performed using Stata 17.0 Software, Revman 5.4 and MedCalc.^1^^,^^2^^,^^3^ MedCalc was a comprehensive statistical analysis software widely used in various medical studies, specifically for receiver operating characteristic (ROC) curve analysis (31). Compared with Stata and Review Manager, MedCalc can control for the influence of heterogeneity within different studies and the limitations of small experimental populations. The effects of heterogeneity were minimized to obtained more accurate results based on DeLong and Binomial exact methods by manually inputting data from each study. Differences with *p* < 0.05 were considered to be statistically significant (32, 33).

## Results

### Search strategy and included studies

**Figure 2:**
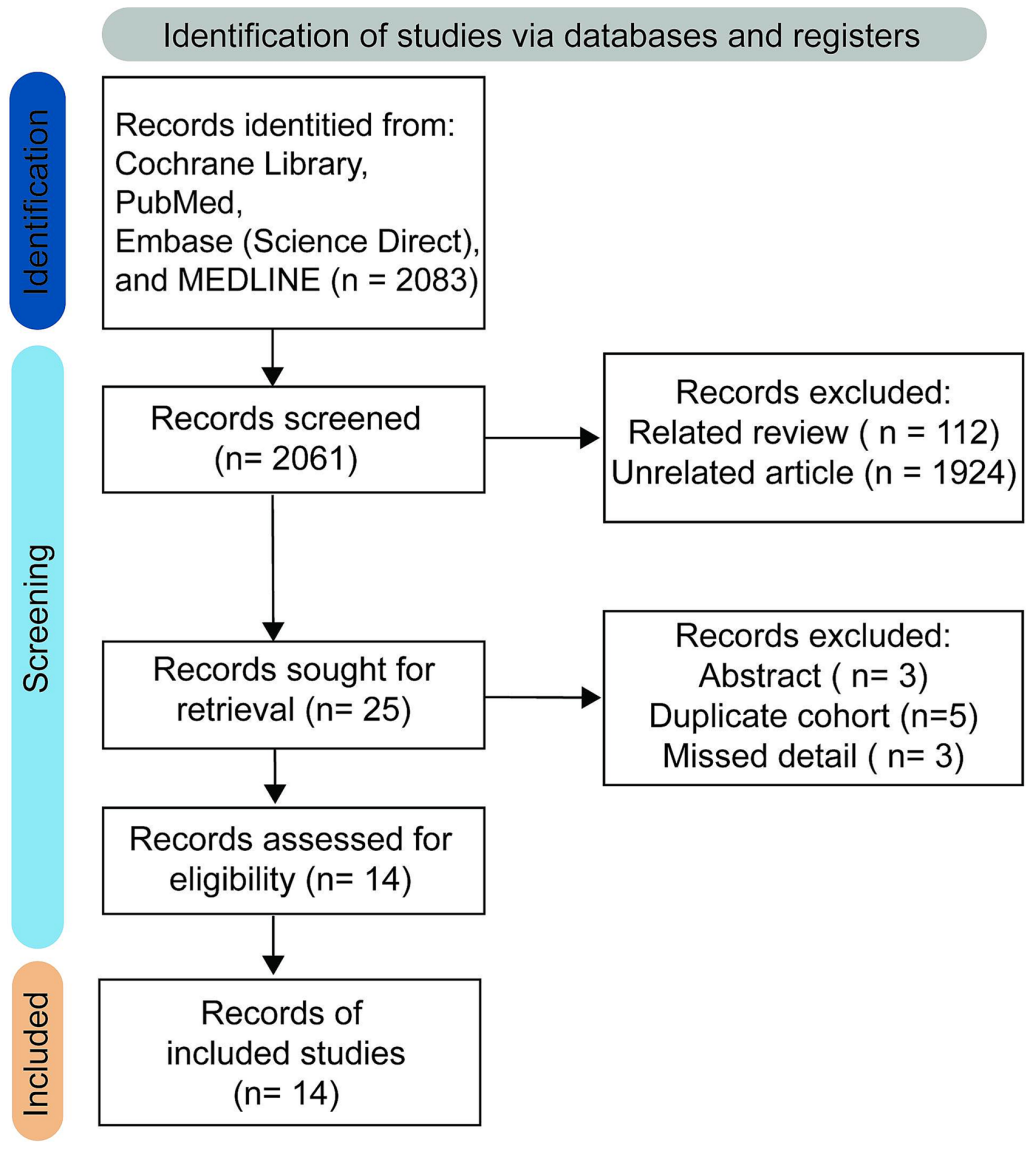
Study selection process.

No restrictions were imposed on study type to maximize the number of studies retrieved. The initial literature search retrieved 2061 studies, of which 112 were identified as relevant reviews, while 1924 were classified as unrelated research papers and were excluded after thorough evaluation. The online database search strategy subsequently identified 25 results, with 14 remaining after excluding 3 abstracts, 5 duplicate cohorts, and 3 studies that lacked essential information. Ultimately, therefore, 14 studies comprising 886 patients fulfilled the inclusion criteria and were included in the qualitative analysis (25, 26, 34–45). A PRISMA flow-diagram illustrating the screening process is presented in Figure 2. Patient characteristics, detailed imaging device parameters, and post-processing approaches are summarized in Tables 2, 3.

**Table 2:** Study details for included articles.

First author^a^	Year^b^	Trial type	Patients number	Mean age (Mean ± SD)	WHO criteria	Grade^c^	Imager vendor^d^	The number of *b*-value^e^	Maximal *b* value	Repetition/echo time^e^	The number of diffusion directions
Hempel JM_a	2017	Prospective	77	51 ± 15	2016	0,29,26,19	Biograph mMR	6	2,500	5900/95	30
Guo H	2022	Prospective	62	52 ± 13	2016	0,14,20,28	MAGNETOM Skyra	10	3,000	4500/111	62
Zeng S	2023	Retrospective	70	47 ± 12	2021	0,19,11,39	MAGNETOM Skyra	11	3,000	3700/72	N/A
Hempel JM_b	2016	Prospective	61	50 ± 14	2007	0,25,15,10	Biograph mMR	6	2,500	5900/95	30
Zhu H	2023	Retrospective	81	47 ± 11	2016	0,28,17,36	Discovery MR750	4	2,500	6500/85	25
Wang X	2020	Prospective	54	48 ± 15	N/A	LGG:19; HGG:32	MAGNETOM Skyra	6	2,500	3000/109	30
Tan Y_a	2019	Retrospective	58	49 ± 7	2016	0,24,15,19	GE Signa HDxt	3	2000	6500/11	30
Qiu J	2023	Prospective	40	55 ± 12	N/A	0,0,14,26	Discovery MR750	3	2,500	N/A	60
Tan Y_b	2020	Retrospective	62	50 ± 13	2016	LGG:26 HGG:36	GE Signa HDxt	3	2000	6500/85	30
Zhao J	2019	Prospective	52	45 ± 12	2016	0,24,8,20	MAGNETOM Verio	3	2000	5500/85	30
Wang P	2023	Prospective	67	50 ± 12	2021	0,22,20,25	MAGNETOM Skyra	3	2000	4200/101	N/A
Xu Z	2021	Retrospective	51	46 ± 15	N/A	0,13,17,21	GE Signa HDxt	3	2000	10,000/97	25
Hempel JM_c	2017	Retrospective	60	51 ± 15	2016	0,28,20,12	Biograph mMR	6	2,500	5900/95	30
Xie Y	2021	Prospective	91	47 ± 7	2016	0,27,20,44	Discovery MR750	3	2,500	6500/85	25

Some detail were not be shown due make the table more clear. ^a^Articles published by the same first author are distinguished using an underscore followed by a letter; ^b^Year of publication; ^c^WHO Glioma Grade I, II, III, IV; ^d^All scanners in published articles were 3.0 Tesla; ^e^*b*-value units in sec/mm^2^; Repetition/Echo Time in msec.

**Table 3:** Diffusion metrics by glioma genotype for included articles.

First author	Mean kurtosis (Mean ± SD, patient populations)				Mean diffusivity (Mean ± SD, patient populations)			
	*IDH* Mut./Wt.	*ATRX*_Del./Exp.	*LOH 1p/19q*_Y/N	*MGMT*_Me./Un.	*IDH* Mut./Wt.	*ATRX*_Del./Exp.	*LOH 1p/19q*_Y/N	*MGMT*_Me./Un.
Hempel JM_a	0.48 ± 0.11;47 0.71 ± 0.12;30	0.41 ± 0.07;25 0.64 ± 0.13;52	0.55 ± 0.09;22 0.58 ± 0.18;55		1.47 ± 0.38;47 1.44 ± 0.26;30	1.72 ± 0.29;25 1.33 ± 0.28;52	1.19 ± 0.25;22 1.57 ± 0.31;55	N/A
Guo H et al.	0.72 ± 0.19;18 0.70 ± 0.17;44				1.32 ± 0.36;18 1.46 ± 0.43;44			
Zeng S et al.	0.56 ± 0.17;35 0.69 ± 0.14;35							
Hempel JM_b	0.43 ± 0.09;33 0.57 ± 0.10;16	0.41 ± 0.11;19 0.51 ± 0.10;26	0.47 ± 0.05;12 0.46 ± 0.13;23	0.50 ± 0.11;19 0.48 ± 0.14;15	1.82 ± 0.37;33 1.43 ± 0.39;16	1.90 ± 0.45;19 1.56 ± 0.29;26	1.63 ± 0.25;12 1.74 ± 0.45;23	1.65 ± 0.33;19 1.57 ± 0.48;15
Zhu H et al.^#^	0.51 ± 0.16;42 0.67 ± 0.18;39							
Wang X	0.55 ± 0.09;17 0.68 ± 0.16;34	0.60 ± 0.14;19 0.60 ± 0.15;32		0.65 ± 0.15;22 0.63 ± 0.15;29	1.69 ± 0.13;17 1.53 ± 0.27;34	1.58 ± 0.21;19 1.56 ± 0.26;32		1.52 ± 0.17;22 1.60 ± 0.28;29
Tan Y_a	0.48 ± 0.16;27 0.67 ± 0.13;31				1.49 ± 0.41;27 1.22 ± 0.26;31			
Qiu J	0.67 ± 0.11;11 1.53 ± 0.25;29				1.42 ± 0.06;11 0.93 ± 0.08;29			
Tan Y_b	0.48 ± 0.15;30 0.66 ± 0.14;32			0.56 ± 0.18;46 0.62 ± 0.14;16	0.90 ± 0.23;30 0.76 ± 0.24;32			0.85 ± 0.26;46 0.78 ± 0.17;16
Zhao J	0.53 ± 0.05;28 0.69 ± 0.06;23				1.49 ± 0.10;28 1.34 ± 0.11;23			
Wang P	0.57 ± 0.11;42 0.76 ± 0.13;25		0.54 ± 0.11;23 0.60 ± 0.12;17		1.19 ± 0.15;42 0.90 ± 0.13;25		1.20 ± 0.14;23 1.17 ± 0.17;17	
Xu Z	0.43 ± 0.15;25 0.63 ± 0.17;26				1.85 ± 0.34;25 1.47 ± 0.40;26			
Hempel JM_c	0.34 ± 0.09;22 0.56 ± 0.10;22							
Xie Y	0.59 ± 0.13;42 0.80 ± 0.08;49				0.89 ± 0.36;42 0.64 ± 0.09;49			

IDH Mut./Wt., IDH Mutation or IDH wild type; ATRX_Del./Exp., ATRX Deletion or ATRX expression; LOH 1p/19q_Y/N, The loss of heterozygosity was/was not happened on Chromosome 1q and 19q; MGMT_Me./Un., the MGMT gene existed/did not appear methylated; SD, Standard Deviation.

# The MK value of Zhu H et al. calculated by the DIPY Toolbox (https://www.dipy.org).

### Assessment of quality and risk of bias

To determine whether the patients involved could be considered as part of the same population, the retrospective studies were classified according to their associated risks. Assessment according to The QUADAS-2 tool indicated a moderate risk of bias in 3 studies due of the type of clinical trial being conducted (36, 38, 45). Furthermore, the randomization process and outcome assessment in these 3 studies may have been influenced, prompting them to be categorized as “having concerns” in domains D1 and D4. Based on quality and risk of bias assessment, it was concluded that these studies could be included in the meta-analysis. No additional bias was identified according to the QUADAS-2 criteria.

### Mean difference in MK and MD among various glioma genotypes

**Figure 3:**
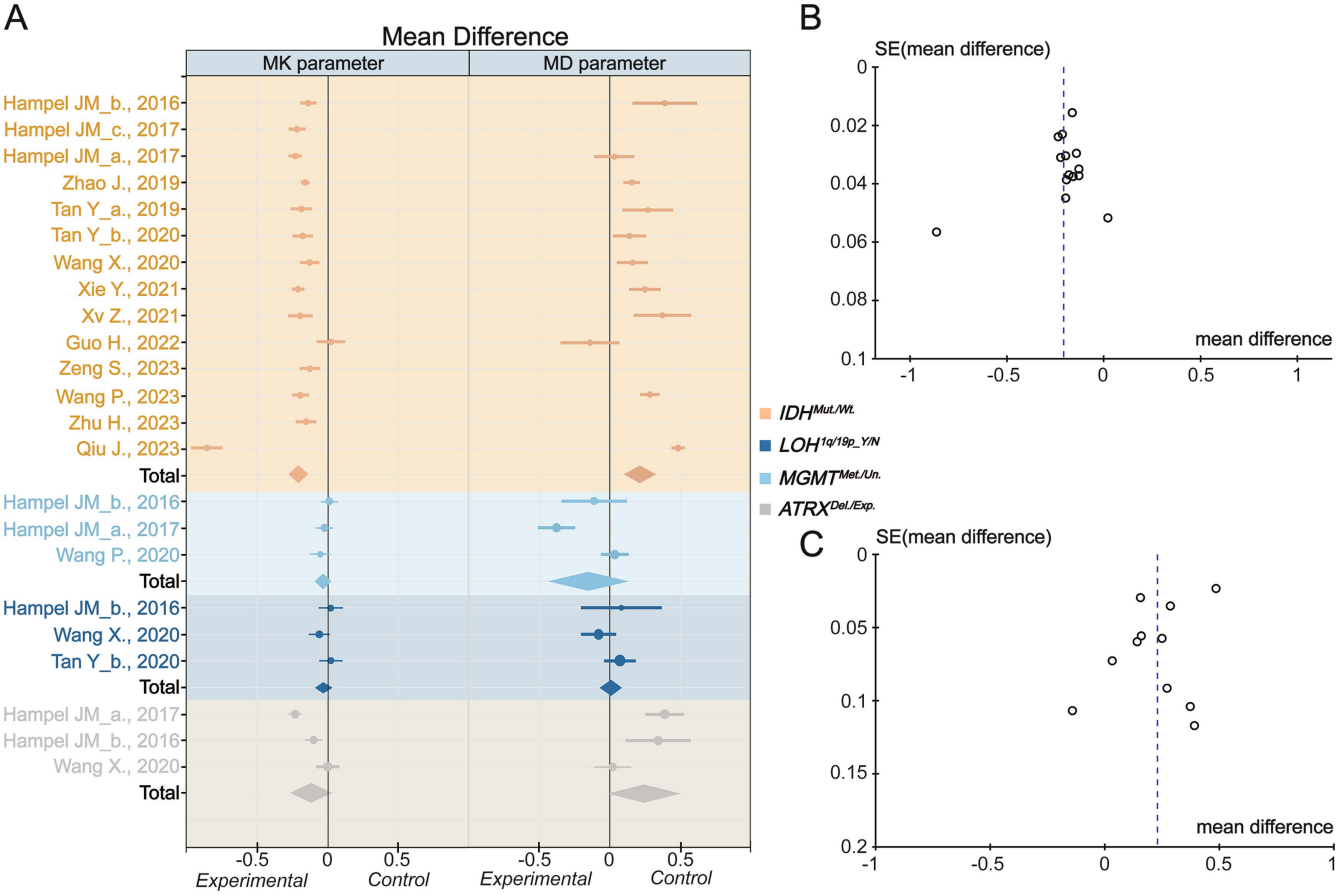
Inverse variance meta-analysis. **(A)** The mean difference for Mean Kurtosis and Mean Diffusivity in various genotypes. **(B)** The funnel plot of Mean Kurtosis. **(C)** The funnel plot of Mean Diffusivity. *IDH_*mut., *IDH* mutation type; *IDH_*wt., *IDH* wild type; LOH, Loss of Heterozygosity; Y, yes; N, no; *MGMT_Me*., *MGMT* methylation; *MGMT_Un.*, *MGMT* Unmethylation; *ATRX_*Del., *ATRX* deletion; *ATRX*_Exp., ATRX expression. The Mean Kurtosis value of Zhu H et al. calculated by the DIPY Toolbox.

Based on the inverse variance random effects model, the mean difference in MK between the *IDH* mutant and wild genotype was −0.21 (95% CI −0.27 to −0.15; *p* < 0.001). The mean difference in MD between the *IDH* mutation and wild type was 0.22 (95% CI 0.11 to 0.33; *p* < 0.001) (Figure 3A). In the random effects model, the pooled mean differences in MK and MD exhibited high heterogeneity (*I*^2^ = 93 and 92%, respectively) (Supplementary Table S9). This means that the DKI parameter could significantly reflect the difference in molecular water conditions between patients with IDH mutation(s) and those with wild-type glioma. However, neither MK nor MD effectively reflected *1p/19q* or *MGMT* expression. It is important to note that, although assessment of the MK prediction value based on *ATRX* status yielded disappointing results, MD exhibited a potential trend toward distinguishing *ATRX* expression status in the meta-analysis (mean difference 0.24 [95% CI −0.01 to 0.50]; *p* = 0.06), which may need further exploration using larger experimental sample sizes. To explore the source of heterogeneity in the mean difference related to *IDH*, funnel plot analysis was performed to evaluate publication bias (Figures 3B,C). The plot reveals clear asymmetry and the presence of an outlier study for MK. However, the analysis indicated that publication bias for MD was minimal. Further analyses will be conducted to investigate heterogeneity. It should be noted that, due to the limited number of data points for *1p/19q*, *MGMT*, and *ATRX* status, publication bias was not assessed nor was heterogeneity or investigated in these cases.

### Heterogeneity resource and subgroup analysis

Based on inverse variance meta-analysis, it was essential to explore the heterogeneity of the mean difference in *IDH* genotypes. The heterogeneity of MK was lower in studies using specific imaging device parameters, including diffusion encoding direction ≤30 (*I*^2^ ≤ 0.5), maximal *b* value <2,500 s/mm^2^ (*I*^2^ = 0), repetition time (TR) ≥ 6,000 ms (*I*^2^ = 0), and echo time (TE) < 100 ms (*I*^2^ = 0.37). Similar outcomes were observed in the MK heterogeneity analysis (Table 4). Although the mean differences in MK and MD decreased slightly after subgroup analysis, their diagnostic utilities remained effective and stable in differentiating between IDH mutant and wild-type genotypes. However, due the low mean differences and limited number of studies, subgroup analyses were not performed for mean differences in the 1p/19q, ATRX, and MGMT genotypes.

**Table 4:** Subgroup analyses of heterogeneity in prediction of IDH status.

Parameter	Subgroup of studies	The number of Studies	The number of patients	*IDH* status		Effect Estimate	*τ*^2^ value	*χ*^2^ value	*Ι*^2^ value	*Z* value (*p* value)
				Mut.	Wild					
MK	Diffusion-encoding Direction = 25	3	223	114	109	−0.20 [−0.23, −0.16]	0	1.66	0	10.95 (*p* < 0.01)
	Diffusion-encoding Direction = 30	7	392	204	188	−0.18 [−0.21, −0.15]	0	12.05	0.5	11.89 (*p* < 0.01)
	Diffusion-encoding Direction > 60	2	99	26	73	−0.42 [−1.28, −0.44]	0.39	132.49	0.99	0.95 (*p* = 0.34)
	Maximal b value < 2,500 s/mm^2^	5	289	152	137	−0.17 [−0.20, −0.15]	0	1.56	0	14.61 (*p* < 0.01)
	Maximal b value = 2,500 s/mm^2^	7	433	214	219	−0.27 [−0.39, −0.16]	0.02	145.83	0.96	4.71 (*p* < 0.01)
	Maximal b value > 2,500 s/mm^2^	2	132	53	79	−0.06 [−0.21, 0.09]	0.01	5.56	0.82	0.79 (*p* = 0.43)
	b value < 6	8	501	247	254	−0.26 [−0.36, −0.17]	0.02	144.61	0.95	5.32 (*p* < 0.01)
	b value ≥ 6	6	353	172	181	−0.15 [−0.21, −0.09]	0	26.90	0.81	4.70 (*p* < 0.01)
	Repetition time < 6,000 msec	8	471	242	229	−0.16 [−0.20, −0.12]	0	27.88	0.75	7.72 (*p* < 0.01)
	Repetition time ≥ 6,000 msec	5	343	166	177	−0.19 [−0.22, −0.16]	0	1.85	0.00	12.95 (*p* < 0.01)
	Echo time < 100 msec	10	634	331	303	−0.18 [−0.21, −0.16]	0	14.31	0.37	15.72 (*p* < 0.01)
	Echo time ≥ 100 msec	3	180	77	103	−0.11 [−0.22, 0.00]	0.01	13.03	0.85	1.94 (*p* = 0.05)
MD	Diffusion-encoding Direction = 25	2	142	67	75	0.28 [0.17, 0.39]	0	1.09	0.08	5.19 (*p* < 0.01)
	Diffusion-encoding Direction = 30	6	348	182	166	0.16 [0.10, 0.23]	0	8.53	0.41	4.94 (*p* < 0.01)
	Diffusion-encoding Direction > 60	2	102	29	73	0.18 [−0.43, 0.79]	0.19	32.54	0.97	0.58 (*p* = 0.56)
	Maximal b value < 2,500 s/mm^2^	5	289	152	137	0.23 [0.14, 0.31]	0.01	12.06	0.67	5.43 (*p* < 0.01)
	Maximal b value = 2,500 s/mm^2^	4	308	150	158	0.26 [0.07, 0.45]	0.04	62.56	0.94	2.71 (*p* = 0.01)
	The number of b value < 6	7	420	205	215	0.28 [0.15, 0.40]	0.03	90.62	0.93	4.32 (*p* < 0.01)
	The number of b value ≥ 6	4	239	115	124	0.11 [−0.07, 0.28]	0.02	13.17	0.77	1.21 (*p* < 0.01)
	Repetition time < 6,000 msec	6	357	185	172	0.15 [0.05, 0.26]	0.01	25.80	0.81	2.96 (*p* < 0.01)
	Repetition time ≥ 6,000 msec	4	262	124	138	0.24 [0.15, 0.32]	0	4.46	0.33	5.25 (*p* < 0.01)
	Echo time < 100 msec	7	439	232	207	0.20 [0.13, 0.28]	0.01	14.36	0.58	5.19 (*p* < 0.01)
	Echo time ≥ 100 msec	3	180	77	103	0.13 [−0.06, 0.32]	0.02	15.79	0.87	1.30 (*p* = 0.19)

### Bivariate model

**Figure 4:**
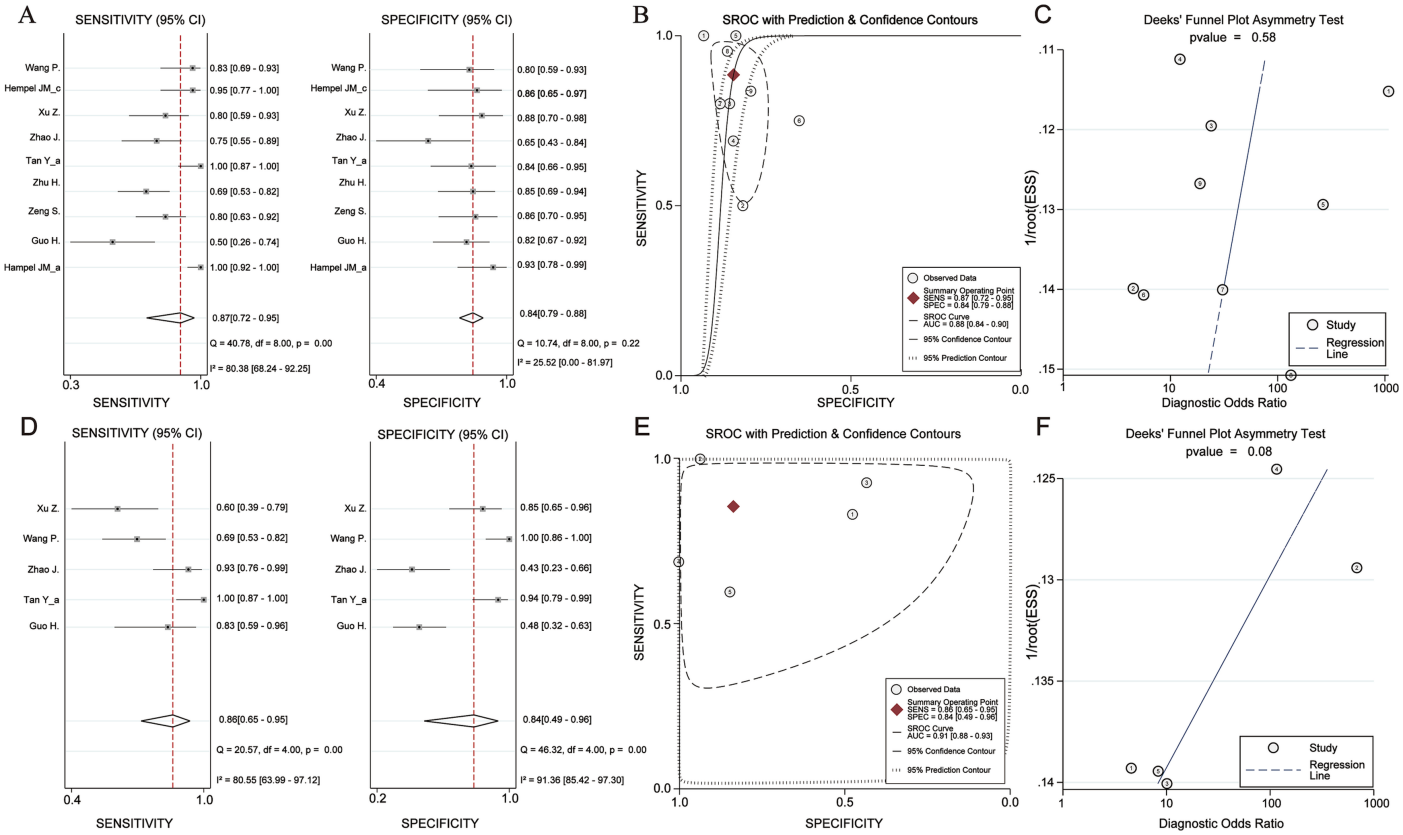
Diagnosis efficacy analysis of Mean Kurtosis and Mean Diffusivity basing on *IDH* status. **(A)** Sensitivity and Specificity effect. **(B)** Receiver operating characteristic curve with Prediction & Confidence Contours. **(C)** The Deek’s funnel plot. **(D)** Sensitivity and Specificity effect. **(E)** Receiver operating characteristic curve with Prediction & Confidence Contours. **(F)** The Deek’s funnel plot.

**Figure 5:**
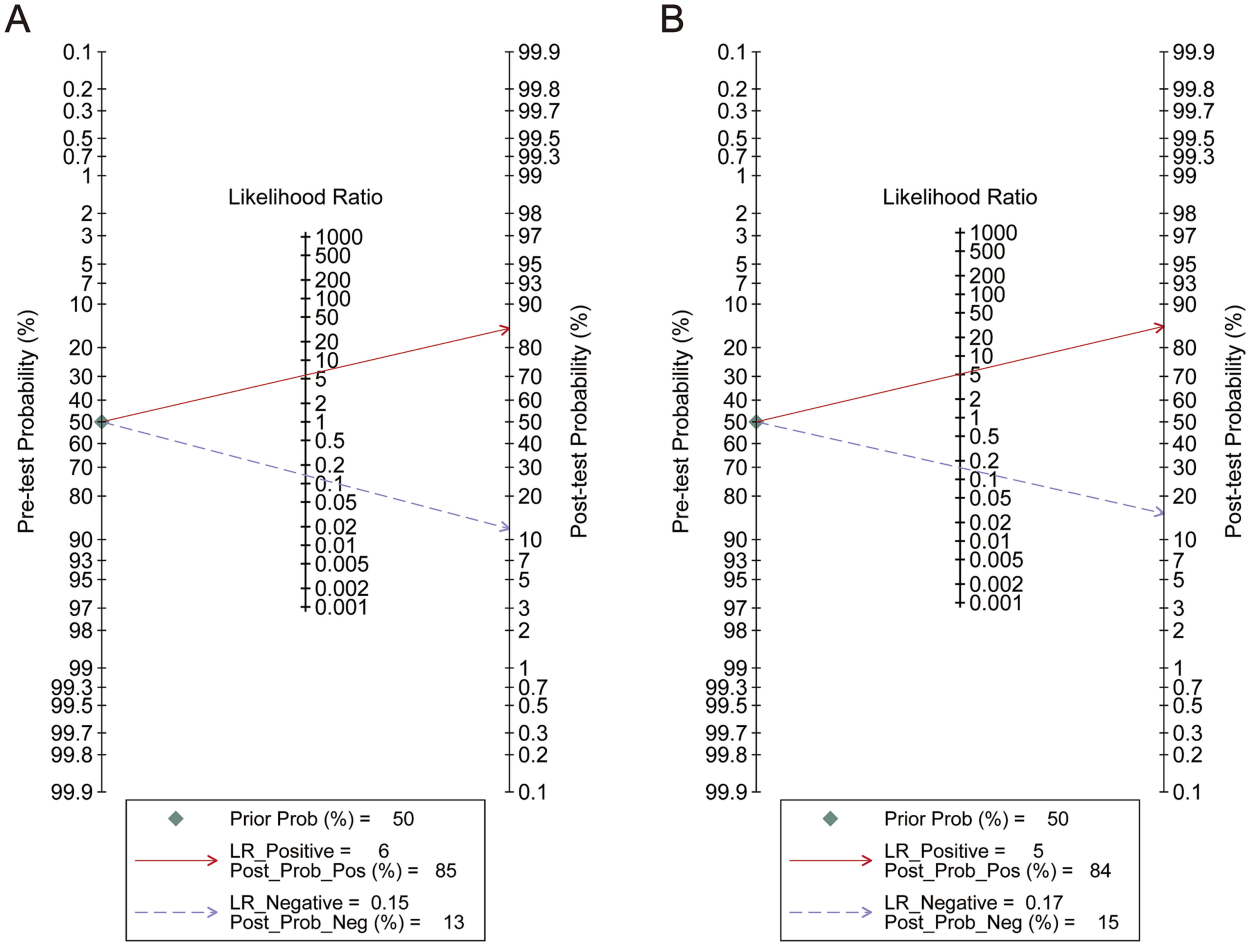
Analysis of pre-test probability. **(A)** The analysis of Mean Kurtosis. **(B)** The analysis of Mean Diffusivity.

**Figure 6:**
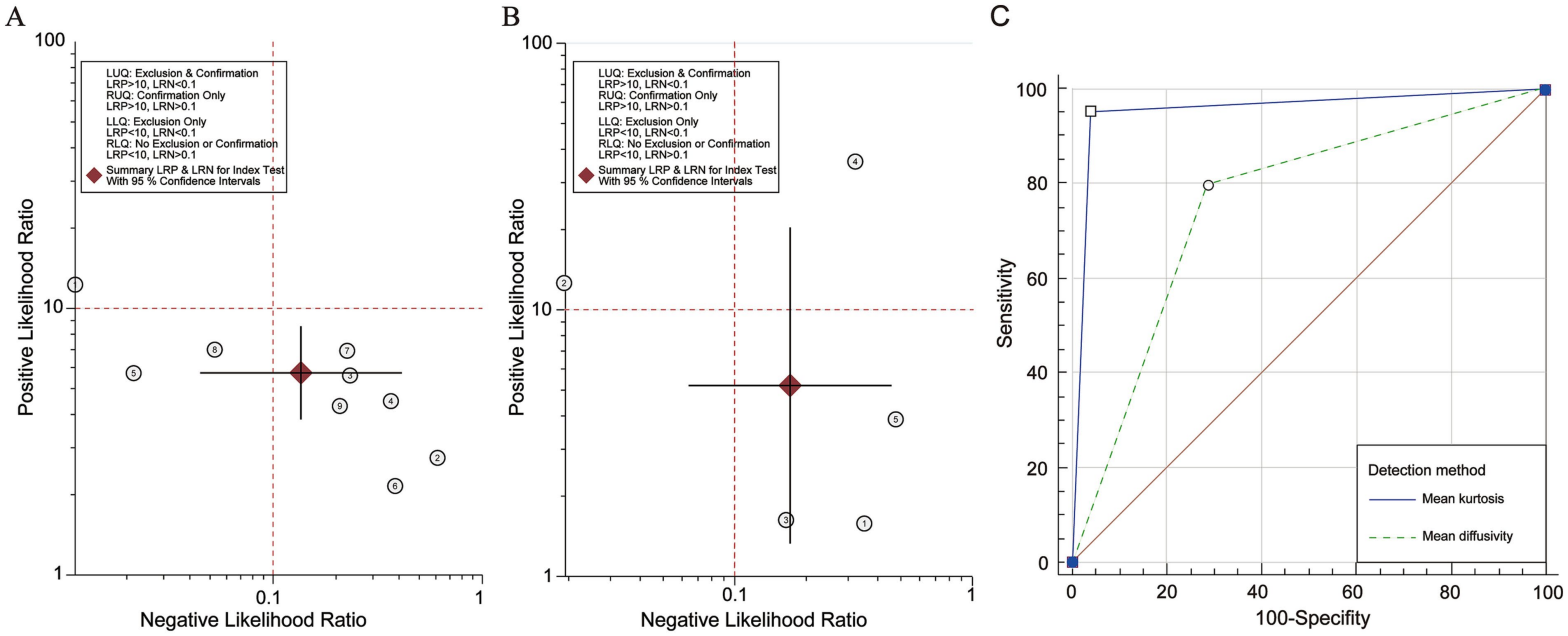
Likelihood ratio scatter gram and summarized Receiver Operating Characteristic analysis of Mean Kurtosis and Mean Diffusivity. **(A)** The likelihood ratio scatter gram of Mean Kurtosis. **(B)** The likelihood ratio scatter gram of Mean Diffusivity. **(C)** The re-calculation in MedCalc.

To perform a bivariate restricted maximum likelihood meta-analysis, some studies that reported the necessary data for diagnostic meta-analysis, such as true positives, false positives, true negatives, false negatives, and area under the curve, were considered (25, 26, 34, 36, 37, 41, 42, 44, 45). When MK was used for predicting IDH status, the summary sensitivity and specificity were found to be 0.87 and 0.84, respectively (Figure 4A). The summary ROC (sROC) curve is presented in Figure 4B with an area under the curve of 0.88. Fagan’s nomogram indicated that individuals who tested positive for MK had an 85% probability of harboring the IDH mutant genotype (Figure 5A). The PLR and NLR were calculated to be 5.51 and 0.15, respectively (Supplementary Figure S1C). Furthermore, the sensitivity and specificity for MD prediction were 0.86 and 0.84 (Figure 4D), respectively, with an area under the sROC curve of 0.91 (Figure 4E). The PLR and NLR for MD were 5.21 and 0.17 (Supplementary Figure S1D). Fagan supported that the patients identified using MD would have an 84% likelihood of having *IDH* mutant gliomas after pathological examination (Figure 5B). The diagnostic scores and odds ratios supported the strong diagnostic value of both MK and MD (Supplementary Figure S1). Deek’s funnel plot indicated low heterogeneity for both MK and MD (Figures 4C,F). Bivariate meta-regression analysis revealed that factors, such as age, publication year, number of patients, percentage of LGG, *b* value, maximal *b* value, TR, TE, direction, and imaging vendor, did not significantly influence prediction accuracy (Table 5). Compared with the pooled sensitivity and specificity, the diagnostic values for MK and MD were inconsistent in terms of the area under the sROC curve. This discrepancy may be attributed to the inadequate curve fitting resulting from the small sample size and limited number of studies (Figures 6A,B). To obtain a more accurate result, MedCalc software was used to recalculate the areas under the sROC curve for the predictive values of MK and MD, which yielded values of 0.96 and 0.76, respectively (Figure 6C).

**Table 5:** Meta-regression analysis of diffusion kurtosis imaging prediction accuracy.

DKI parameter	Characteristics	Coef.	Std. err.	*t*	*p* > |t|	95% CI	
MK	Age	< −0.01	0.20	−0.02	0.99	−0.55	0.55
	Publish year	−0.18	0.24	−0.65	0.55	−0.83	0.52
	The number of Patients	0.001	0.05	0.02	0.98	−0.13	0.13
	LGG percent	3.10	5.42	0.57	0.62	−11.96	18.18
	The number of b value	−0.02	0.15	−0.10	0.93	−0.44	0.41
	Maximal b value	< −0.01	< 0.01	−0.65	0.55	< −0.01	< 0.01
	Repetition time	0	0	0.66	0.55	< −0.01	< 0.01
	Echo time	−0.02	0.04	−0.51	0.64	−0.13	0.09
	The number of (diffusion) directions	−0.04	0.04	−1.07	0.36	−0.15	0.08
	Vendor A	2.49	1.38	1.81	0.15	−1.33	6.30
	Vendor B	−1.14	0.81	−1.41	0.23	−3.40	1.11
	Vendor C	−0.28	1.29	−0.17	0.88	−3.80	3.37
	Vendor D	0.92	1.32	0.70	0.52	−2.75	4.60
MD	Age	−0.09	0.17	−0.55	0.68	−2.26	2.07
	Publish year	0.15	0.24	0.60	0.66	−2.96	3.25
	The number of Patients	−0.03	0.04	−0.70	0.61	−0.49	0.44
	LGG percent	−1.37	5.92	−0.23	0.86	−76.65	73.91
	The number of b value	−0.22	0.29	−0.76	0.59	−3.94	3.50
	Maximal b value	< −0.01	< 0.01	−0.76	0.59	−0.02	0.02
	Repetition time	0	0	−0.76	0.58	< −0.01	< 0.01
	Echo time	< −0.01	0.01	−0.48	0.72	−0.15	0.14
	The number of (diffusion) directions	−0.05	0.17	−0.31	0.81	−2.26	2.15
	Vendor A	−0.67	0.88	−0.76	0.59	−11.83	10.49
	Vendor B	−	−	−	−	−	−
	Vendor C	−	−	−	−	−	−
	Vendor D	0.67	0.86	0.76	0.59	−10.49	11.83

To avoid rater bias, MRI vendors were anonymized. For example, Vendor A was Biograph brand; Vendor B: Magnetom brand; Vendor C: Discovery brand; Vendor D: GE Signa brand. In the meta-regression analysis of MD, the Vendor B and C only was involved in the single included article. Thus, the influence of Vendor B and C could not be analyzed in the MD.

## Discussion

The present meta-analysis included 14 clinical studies comprising 886 patients to evaluate the predictive value of DKI for the molecular classification of gliomas. Our findings indicated that MD and MK, as DKI parameters, could effectively distinguish specific molecular subtypes. Specifically, higher MK and lower MD values were associated with *IDH* status, underscoring the utility of these parameters for molecular prediction. The summarized sROC curve and meta-regression analyses, which accounted for relevant covariates, further confirmed the efficacy and stability of DKI parameters for predicting *IDH* status. Subgroup analyses provided insights into the sources of heterogeneity and the impact of imaging protocol adjustments. We found that TR, TE, maximal *b*-value, and diffusion-encoding direction played significant roles in reducing heterogeneity. A longer TR permits adequate longitudinal relaxation (T1) and signal recovery, thereby minimizing the influence of T1 effects on the diffusion parameters (46). A shorter TE reduces signal attenuation, yielding more stable measurements (47). Excessively low maximal *b*-values were found to improve signal quality, leading to more stable estimates of MK and MD (48). Although a lower diffusion direction could regulate heterogeneity, we considered a trade-off between heterogeneity, prediction value, and signal quality. Based on the *I*^2^ value and effect estimates from the subgroup analysis of heterogeneity, we inferred that, although a greater number of diffusion directions may lead to heterogeneity across different studies and patient populations, it also captured more complex and detailed information about water molecule movement. These enhanced data are valuable for predicting the genetic status of gliomas and for personalized medical treatment. Therefore, we recommend improving the quality of MRI images and reducing heterogeneity by adjusting scanning parameters, such as scan time, magnetic field intensity, and imaging sequence. The utility of DKI in predicting glioma molecular expression can be further improved by appropriately increasing the number of diffusion directions (49).

From a macroscopic physical structure perspective, as an imaging technique, DKI can detect diffusion information based on a non-Gaussian diffusion model and compute signal differences along several gradient directions to obtain a detailed signal that measures water diffusion (50). As critical parameters, MK and MD can capture and reflect different motion states of water molecules within diverse organizational structures. MK is typically associated with structural complexity and tends to increase when the diffusion pathways of water molecules become more intricate due to high cell density, cellular atypia, and alterations in the extracellular matrix. In contrast, the MD value reflects the degree of diffusion of all water molecules in each direction, indicating the diffusion ability of water molecules in tissues. A higher MD value represents an increase in the overall water molecule diffusion and a decrease in diffusion resistance (51–53). Furthermore, genetic status can influence tumor size, growth direction, texture, and blood supply, all of which can affect the diffusion of water molecules. These factors contribute to the predictive value of DKI parameters in determining molecular type.

From a genetic and microstructural perspective, previous studies have demonstrated that genetic expression status, particularly regarding *IDH*, can significantly influence cell metabolism and epigenetic changes (54, 55). For example, *IDH*-mutant gliomas are associated with the accumulation of metabolites, such as 2-hydroxyvalerate, which can elevate intracellular oxidative stress levels (56). *IDH* mutations inhibit histone demethylation and cell differentiation (57). However, wild-type *IDH* tumor tissue exhibited the opposite effect. These specific genetic statuses may influence the proliferative ability and the DKI characteristics of gliomas. Zhao et al. conducted further analyses and demonstrated that *IDH* wild-type gliomas exhibited high proliferative activity, which influenced tumor progression and MK and MD values in DKI. More specifically, the movement of water molecules in complex wild-type *IDH* tissues was hindered, leading to a lower diffusion coefficient and, consequently, a reduced MD value. Furthermore, the diffusion trajectory of water molecules was more irregular, causing a deviation from the Gaussian distribution and resulting in a higher MK value (26, 58, 59). These factors may explain why MK and MD can differentiate *IDH* conditions. However, DKI parameters appeared to be less effective in distinguishing specific genetic statuses, such as *1p/19q* codeletion, *MGMT*, and *ATRX*. Based on the biological studies, we found that these genes did not significantly influence the diffusion characteristics of water molecules. For example, the mechanisms underlying *1p/19q* codeletion and *ATRX* primarily regulate genomic stability and chromosomal structural changes (60). Additionally, the methylation status of *MGMT* mainly affects the sensitivity of patients with glioma to chemotherapeutic drugs (61). Thus, genetic alterations do not directly affect the tissue structure or diffusion characteristics of water molecules in tumors, leading to the conclusion that DKI may not accurately reflect the expression status of these genes within tumor structures.

Recent meta-analyses demonstrated that DKI can distinguish the LGG and HGG and provide valuable insights (Table 1). With advances in imaging technology, radiologists and clinicians want to identify whether the predictive utility of DKI in determining the molecular genetic status of gliomas is stable equally. Our analysis suggests that DKI and its parameters, particularly MK and MD, offer some utility in differentiating glioma molecular status. However, a model with stable accuracy has not yet been sufficiently tested. Therefore, we suggest that DKI should be of interest to researchers, and large-scale clinical trials and explorations should be conducted to compare it with other modes to identify which MRI mode yields more stable diagnostic utility. We believe that predictive capabilities will improve gradually in the future, paving the way for advances in the noninvasive molecular diagnosis of gliomas.

Compared with recent meta-analyses, our analysis further supported that DKI can predict the glioma molecular subtypes, especially *IDH* status. However, the present meta-analysis had some limitations. First, although we provided data supporting the utility of DKI parameters in predicting *IDH* status, the related-parameter(s) prediction thresholds remained unclear due to missing patient data and the insufficient sample size. Second, equally important genotyping, such as *1p/19q*, *ATRX*, and *MGMT*, were not analyzed in this meta-analysis due to the absence of relevant information, which could not obtained despite attempts to contact the authors of the studies in question. As such, studies with larger populations are necessary to further investigate these parameters.

## Conclusion

Our findings provide evidence supporting the predictive utility of DKI in glioma molecular classification, particularly regarding *IDH* status. We recommend that DKI and its associated parameters be explored in future clinical trials and compared with other modalities to identify more stable and valuable models. This will improve the accuracy and comprehensiveness of future diagnostic models.

## Data Availability

The original contributions presented in the study are included in the article/Supplementary material, further inquiries can be directed to the corresponding author.

## Footnotes

^1^ https://www.stata-uk.com/software/stata

^2^ https://training.cochrane.org/online-learning/core-software

^3^ https://www.medcalc.org/

## Supplementary material

SUPPLEMENTARY FIGURE S1 The quantification of Diffusion Kurtosis Imaging diagnostic effects. **(A)** The forest plots of diagnostic score, odds ratio in Mean Kurtosis. **(B)** The forest plots of diagnostic score, odds ratio in Mean Diffusivity. **(C)** The forest plots of positive diagnostic likelihood ratio and negative diagnostic likelihood ratio in Mean Kurtosis. **(D)** The forest plots of positive diagnostic likelihood ratio and negative diagnostic likelihood ratio in Mean Diffusivity.
