# Supplementary figures and images for: The diagnostic and prediction performance of MR diffusion kurtosis imaging in the glioma molecular classification: a systematic review and meta-analysis
Source: Front Neurol. 2025 Apr 25;16:1543619. doi: 10.3389/fneur.2025.1543619 (PMC12061957; doi:10.3389/fneur.2025.1543619)

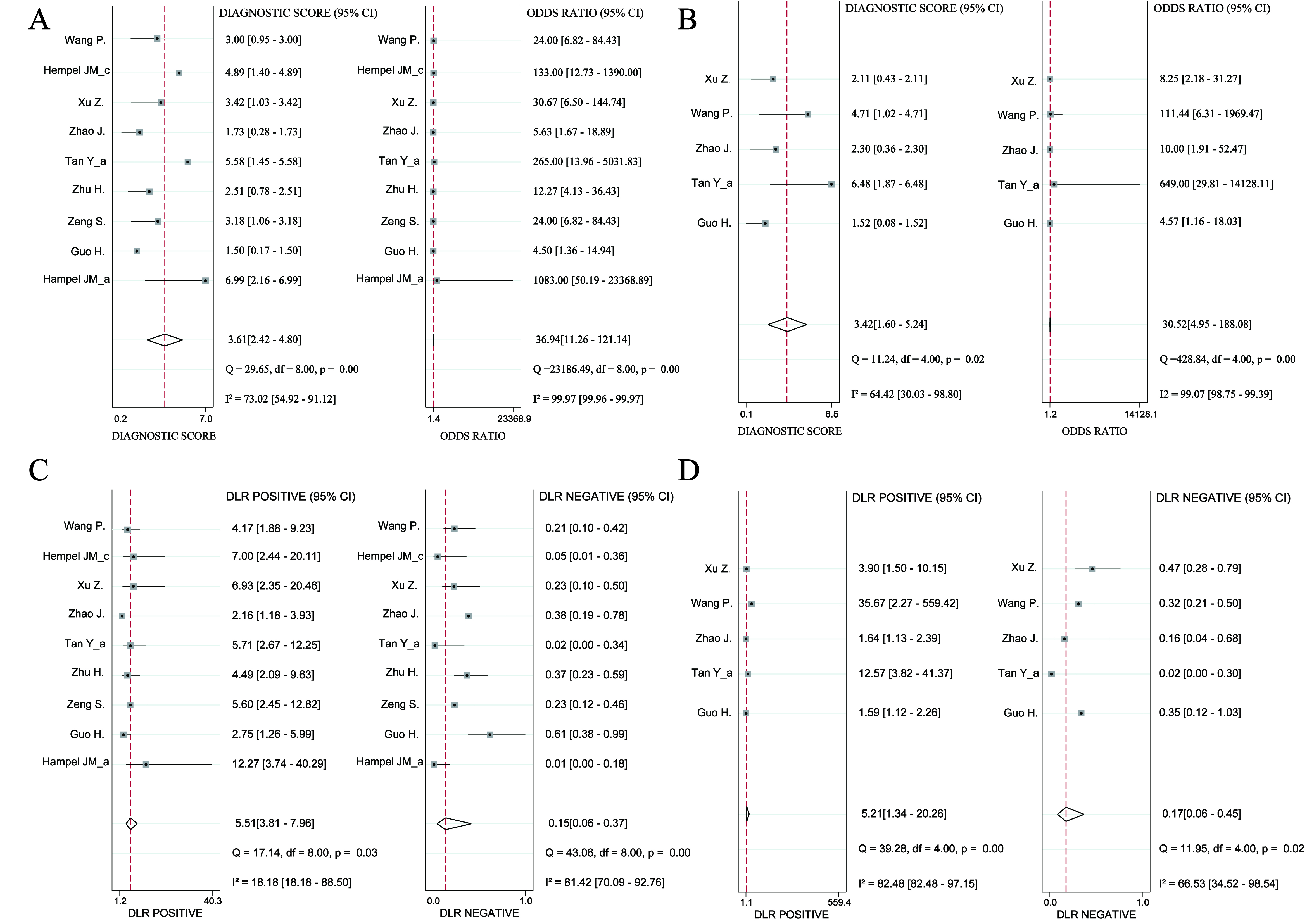

Supplement: SUPPLEMENTARY FIGURE S1 — The quantification of Diffusion Kurtosis Imaging diagnostic effects. (A) The forest plots of diagnostic score, odds ratio in Mean Kurtosis. (B) The forest plots of diagnostic score, odds ratio in Mean Diffusivity. (C) The forest plots of positive diagnostic likelihood ratio and negative diagnostic likelihood ratio in Mean Kurtosis. (D) The forest plots of positive diagnostic likelihood ratio and negative diagnostic likelihood ratio in Mean Diffusivity. [file Image_1.JPEG]
