# Supplementary material for: The diagnostic and prediction performance of MR diffusion kurtosis imaging in the glioma molecular classification: a systematic review and meta-analysis
Source: Front Neurol. 2025 Apr 25;16:1543619. doi: 10.3389/fneur.2025.1543619 (PMC12061957; doi:10.3389/fneur.2025.1543619)
Supplement: Supplementary file 2 [file Table_1.docx]

| **Search Resource** | **Query** | **Sort By** | **Filters** | **Search Details** | **Results** | **Time (UTC+8)** |
| --- | --- | --- | --- | --- | --- | --- |
| Pubmed | (((((((glioma molecular subtype) OR (glioma genotyping)) OR (glioma)) OR (Glioblastoma)) OR (Astrocytoma)) OR (Oligodendroglioma)) AND (DKI)) OR (Diffusion Kurtosis Imaging) | N/A | N/A | (((("glioma"[MeSH Terms] OR "glioma"[All Fields] OR "gliomas"[All Fields] OR "glioma s"[All Fields]) AND ("molecular"[All Fields] OR "moleculars"[All Fields]) AND ("subtype"[All Fields] OR "subtyped"[All Fields] OR "subtypes"[All Fields] OR "subtyping"[All Fields] OR "subtypings"[All Fields])) OR (("glioma"[MeSH Terms] OR "glioma"[All Fields] OR "gliomas"[All Fields] OR "glioma s"[All Fields]) AND ("genotype"[MeSH Terms] OR "genotype"[All Fields] OR "genotypes"[All Fields] OR "genotypic"[All Fields] OR "genotype s"[All Fields] OR "genotyped"[All Fields] OR "genotyper"[All Fields] OR "genotypical"[All Fields] OR "genotypically"[All Fields] OR "genotyping"[All Fields] OR "genotypings"[All Fields] OR "genotypization"[All Fields])) OR ("glioma"[MeSH Terms] OR "glioma"[All Fields] OR "gliomas"[All Fields] OR "glioma s"[All Fields]) OR ("glioblastoma"[MeSH Terms] OR "glioblastoma"[All Fields] OR "glioblastomas"[All Fields]) OR ("astrocytoma"[MeSH Terms] OR "astrocytoma"[All Fields] OR "astrocytomas"[All Fields]) OR ("oligodendroglioma"[MeSH Terms] OR "oligodendroglioma"[All Fields] OR "oligodendrogliomas"[All Fields])) AND "DKI"[All Fields]) OR (("diffusable"[All Fields] OR "diffusant"[All Fields] OR "diffusants"[All Fields] OR "diffuse"[All Fields] OR "diffusely"[All Fields] OR "diffuses"[All Fields] OR "diffusibility"[All Fields] OR "diffusible"[All Fields] OR "diffusion"[MeSH Terms] OR "diffusion"[All Fields] OR "diffused"[All Fields] OR "diffusing"[All Fields] OR "diffusions"[All Fields] OR "diffusive"[All Fields] OR "diffusively"[All Fields] OR "diffusivities"[All Fields] OR "diffusivity"[All Fields]) AND "Kurtosis"[All Fields] AND ("image"[All Fields] OR "image s"[All Fields] OR "imaged"[All Fields] OR "imager"[All Fields] OR "imager s"[All Fields] OR "imagers"[All Fields] OR "images"[All Fields] OR "imaging"[All Fields] OR "imaging s"[All Fields] OR "imagings"[All Fields])) | 1,849 | 2024/7/25/ 18:54:30 |

**Table S1 The search parameters and resource in Pubmed**

**Translations：**

glioma: "glioma"[MeSH Terms] OR "glioma"[All Fields] OR "gliomas"[All Fields] OR "glioma's"[All Fields]

molecular: "molecular"[All Fields] OR "moleculars"[All Fields]

subtype: "subtype"[All Fields] OR "subtyped"[All Fields] OR "subtypes"[All Fields] OR "subtyping"[All Fields] OR "subtypings"[All Fields]

glioma: "glioma"[MeSH Terms] OR "glioma"[All Fields] OR "gliomas"[All Fields] OR "glioma's"[All Fields]

genotyping: "genotype"[MeSH Terms] OR "genotype"[All Fields] OR "genotypes"[All Fields] OR "genotypic"[All Fields] OR "genotype's"[All Fields] OR "genotyped"[All Fields] OR "genotyper"[All Fields] OR "genotypical"[All Fields] OR "genotypically"[All Fields] OR "genotyping"[All Fields] OR "genotypings"[All Fields] OR "genotypization"[All Fields]

glioma: "glioma"[MeSH Terms] OR "glioma"[All Fields] OR "gliomas"[All Fields] OR "glioma's"[All Fields]

Glioblastoma: "glioblastoma"[MeSH Terms] OR "glioblastoma"[All Fields] OR "glioblastomas"[All Fields] OR "glioblastoma's"[All Fields]

Astrocytoma: "astrocytoma"[MeSH Terms] OR "astrocytoma"[All Fields] OR "astrocytomas"[All Fields]

Oligodendroglioma: "oligodendroglioma"[MeSH Terms] OR "oligodendroglioma"[All Fields] OR "oligodendrogliomas"[All Fields]

Diffusion: "diffusable"[All Fields] OR "diffusant"[All Fields] OR "diffusants"[All Fields] OR "diffuse"[All Fields] OR "diffusely"[All Fields] OR "diffuses"[All Fields] OR "diffusibility"[All Fields] OR "diffusible"[All Fields] OR "diffusion"[MeSH Terms] OR "diffusion"[All Fields] OR "diffused"[All Fields] OR "diffusing"[All Fields] OR "diffusions"[All Fields] OR "diffusive"[All Fields] OR "diffusively"[All Fields] OR "diffusivities"[All Fields] OR "diffusivity"[All Fields]

Imaging: "image"[All Fields] OR "image's"[All Fields] OR "imaged"[All Fields] OR "imager"[All Fields] OR "imager's"[All Fields] OR "imagers"[All Fields] OR "images"[All Fields] OR "imaging"[All Fields] OR "imaging's"[All Fields] OR "imagings"[All Fields]
