# Supplementary material for: The diagnostic and prediction performance of MR diffusion kurtosis imaging in the glioma molecular classification: a systematic review and meta-analysis
Source: Front Neurol. 2025 Apr 25;16:1543619. doi: 10.3389/fneur.2025.1543619 (PMC12061957; doi:10.3389/fneur.2025.1543619)
Supplement: Supplementary file 3 [file Table_2.docx]

**Table S2 The search parameters and resource in Embase and Medline**

| **No.** | **Query Results** | **Results** | **Date** |
| --- | --- | --- | --- |
| #11. | #9 AND #10 | 167 | 07/25/24 |
| #10. | #7 OR #8 | 2,726 |  |
| #9. | #1 OR #2 OR #3 OR #4 OR #5 OR #6 | 209,975 |  |
| #8. | 'diffusion kurtosis imaging' | 1,654 |  |
| #7. | 'dki' | 2,209 |  |
| #6. | 'oligodendroglioma' | 12,194 |  |
| #5. | 'astrocytoma' | 37,512 |  |
| #4. | 'glioblastoma' | 109,367 |  |
| #3. | 'glioma' | 123,929 |  |
| #2. | 'glioma genotyping' | 14 |  |
| #1. | 'glioma molecular subtype' | 14 |  |
