# Supplementary material for: The diagnostic and prediction performance of MR diffusion kurtosis imaging in the glioma molecular classification: a systematic review and meta-analysis
Source: Front Neurol. 2025 Apr 25;16:1543619. doi: 10.3389/fneur.2025.1543619 (PMC12061957; doi:10.3389/fneur.2025.1543619)
Supplement: Supplementary file 4 [file Table_3.docx]

**Table S3 The search parameters and resource in Cochrane**

| **Search Resource** | **Query** | **Sort By** | **Filters** | **Search Details** | **Results** | **Time (UTC+8)** |
| --- | --- | --- | --- | --- | --- | --- |
| Cochrane | (((((((glioma molecular subtype) OR (glioma genotyping)) OR (glioma)) OR (Glioblastoma)) OR (Astrocytoma)) OR (Oligodendroglioma)) AND (DKI)) OR (Diffusion Kurtosis Imaging) in Title Abstract Keyword | N/A | N/A | Not Applicable | 22 | 2024/7/25/ 18:02:37 |
