# Supplementary material for: The diagnostic and prediction performance of MR diffusion kurtosis imaging in the glioma molecular classification: a systematic review and meta-analysis
Source: Front Neurol. 2025 Apr 25;16:1543619. doi: 10.3389/fneur.2025.1543619 (PMC12061957; doi:10.3389/fneur.2025.1543619)
Supplement: Supplementary file 5 [file Table_4.docx]

**Table S4 The search parameters and resource in Web of science**

| **Search Resource** | **Quick add keywords** | **Sort By** | **Filters** | **Search Details** | **Results** | **Time (UTC+8)** |
| --- | --- | --- | --- | --- | --- | --- |
| **Web of science** | 1. diffusion kurtosis imaging; 2. diffusional kurtosis imaging;  3. DKI; 4. diffusion kurtosis imaging DKI; 5. mean kurtosis;  6. diffusion kurtosis; 7. histogram analysis; 8. diffusional kurtosis; 9. Non Gaussian diffusion; 10. Intravoxel incoherent motion | N/A | N/A | (((((((glioma molecular subtype) OR (glioma genotyping)) OR (glioma)) OR (Glioblastoma)) OR (Astrocytoma)) OR (Oligodendroglioma)) AND (DKI)) OR (Diffusion Kurtosis Imaging) | 2668 | 2024/7/25/ 17:32:45 |
