# Supplementary material for: The diagnostic and prediction performance of MR diffusion kurtosis imaging in the glioma molecular classification: a systematic review and meta-analysis
Source: Front Neurol. 2025 Apr 25;16:1543619. doi: 10.3389/fneur.2025.1543619 (PMC12061957; doi:10.3389/fneur.2025.1543619)
Supplement: Supplementary file 6 [file Table_5.docx]

**Table S5. QUADAS-2 results for Studies Meeting Search Inclusion Criteria**

| **Study** | **RISK OF BIAS** | | | | **APPLICABILITY CONCERNS** | | |
| --- | --- | --- | --- | --- | --- | --- | --- |
|  | **PATIENT SELECTION** | **INDEX TEST** | **REFERENCE STANDARD** | **FLOW AND TIMING** | **PATIENT SELECTION** | **INDEX TEST** | **REFERENCE STANDARD** |
|  |  |  |  |  |  |  |  |
| Hempel JM_a | L | L | L | L | L | L | L |
| Guo H | L | L | L | L | L | L | L |
| Zeng S | H | L | L | L | L | L | L |
| Hempel JM_b | L | L | L | L | L | L | L |
| Zhu H | L | L | L | L | L | L | L |
| Wang X | L | L | L | L | L | L | L |
| Tan Y_a | L | L | L | L | L | L | L |
| Qiu J | L | L | L | L | L | L | L |
| Tan Y_b | H | L | L | L | L | L | L |
| Zhao J | L | L | L | L | L | L | L |
| Wang P | L | L | L | L | L | L | L |
| Xu Z | L | L | L | L | L | L | L |
| Hempel JM_c | H | L | L | L | L | L | L |
| Xie Y | L | L | L | L | L | L | L |

**Abbreviation:** L: Low Risk; H: High Risk; U: Unclear Risk
