# Supplementary material for: The diagnostic and prediction performance of MR diffusion kurtosis imaging in the glioma molecular classification: a systematic review and meta-analysis
Source: Front Neurol. 2025 Apr 25;16:1543619. doi: 10.3389/fneur.2025.1543619 (PMC12061957; doi:10.3389/fneur.2025.1543619)
Supplement: Supplementary file 7 [file Table_6.docx]

**Table S6. Quality assessment of the included studies’ bias**

| **Study ID** | **D1** | **D2** | **D3** | **D4** | **D5** | **Overall bias** |
| --- | --- | --- | --- | --- | --- | --- |
| Hempel JM_a | Low | Low | Low | Low | Low | Low |
| Guo H | Low | Low | Low | Low | Low | Low |
| Zeng S | Some concerns | Low | Low | Some concerns | Low | Low |
| Hempel JM_b | Low | Low | Low | Low | Low | Low |
| Zhu H | Low | Low | Low | Low | Low | Low |
| Wang X | Low | Low | Low | Low | Low | Low |
| Tan Y_a | Low | Low | Low | Low | Low | Low |
| Qiu J | Low | Low | Low | Low | Low | Low |
| Tan Y_b | Some concerns | Low | Low | Some concerns | Low | Low |
| Zhao J | Low | Low | Low | Low | Low | Low |
| Wang P | Low | Low | Low | Low | Low | Low |
| Xu Z | Low | Low | Low | Low | Low | Low |
| Hempel JM_c | Some concerns | Low | Low | Some concerns | Low | Low |
| Xie Y | Low | Low | Low | Low | Low | Low |

D1: Bias arising from the randomization process; D2: Bias due to deviations from intended interventions;

D3: Bias due to missing outcome data; D4: Bias in measurement of the outcome; D5: Bias in selection of the reported results

Low risk of bias: The study is judged to beat low risk of bias for all domains for this result.

Some concerns: The study is judged to raise some concerns in at least one domain for this result, but not to beat high risk of bias for any domain.

High risk of bias: The study is judged to beat high risk of bias in at least one domain, or to have some concerns for multiple domains in away that substantially lowers confidence in the result.
