# Supplementary material for: The diagnostic and prediction performance of MR diffusion kurtosis imaging in the glioma molecular classification: a systematic review and meta-analysis
Source: Front Neurol. 2025 Apr 25;16:1543619. doi: 10.3389/fneur.2025.1543619 (PMC12061957; doi:10.3389/fneur.2025.1543619)
Supplement: Supplementary file 8 [file Table_7.docx]

| **Parameter** | **Subgroup** | **Studies** | **Participants** | **Statistical Method** | **Effect Estimate** | ***I*^2^** |
| --- | --- | --- | --- | --- | --- | --- |
| MK | IDH^mut.^ Vs IDH^wt.^ | 14 | 863 | Random model | -0.21 [-0.27, -0.15] | 93% |
|  | LOH_1p/19q Y vs N | 3 | 152 | Fixed model | -0.02 [-0.06, 0.02] | 0% |
|  | MGMT^Methy.^ vs MGMT^Unmethy.^ | 3 | 147 | Random model | 0 [-0.05, 0.05] | 5% |
|  | ATRX^Del.^ vs ATRX^Exp.^ | 3 | 173 | Random model | -0.11 [-0.25, 0.02] | 93% |
| MD | IDH^mut.^ Vs IDH^wt.^ | 12 | 740 | Random model | 0.23 [0.13, 0.33] | 92% |
|  | LOH_1p/19q Y vs N | 3 | 152 | Random model | -0.15 [-0.44, 0.14] | 92% |
|  | MGMT^Methy.^ vs MGMT^Unmethy.^ | 3 | 147 | Random model | 0.01 [-0.07, 0.09] | 40% |
|  | ATRX^Del.^ vs ATRX^Exp.^ | 3 | 173 | Random model | 0.24 [-0.01, 0.50] | 87% |

**Table S7 The summary of pooled mean differences in the each molecular subgroup**

**Abbreviation:** IDH^mut.^: IDH mutation type; IDH^wt.^: IDH wild type; LOH: Loss of Heterozygosity; Y: yes; N: no; MGMT^Methy.^: MGMT methylation; MGMT^Unmethy.^: MGMT Unmethylation; ATRX^Del.^: ATRX deletion; ATRX^Exp.^: ATRX expression;
